# A Case of Suspected Hyperphenylalaninemia at Newborn Screening by Tandem Mass Spectrometry during Total Parenteral Nutrition

**DOI:** 10.3390/metabo10020044

**Published:** 2020-01-24

**Authors:** Damiana Pieragostino, Ilaria Cicalini, Silvia Di Michele, Paola Fusilli, Giovanna Cotugno, Rossella Ferrante, Ines Bucci, Carlo Dionisi-Vici, Liborio Stuppia, Vincenzo De Laurenzi, Claudia Rossi

**Affiliations:** 1 Center for Advanced Studies and Technology (CAST), University “G. d’Annunzio” of Chieti-Pescara, 66100 Chieti, Italy; dpieragostino@unich.it (D.P.); ilaria.cicalini@unich.it (I.C.); rossellaferrante@yahoo.it (R.F.); ibucci@unich.it (I.B.); stuppia@unich.it (L.S.); delaurenzi@unich.it (V.D.L.); 2 Department of Medical, Oral and Biotechnological Sciences, University ‘‘G. d’Annunzio’’ of Chieti-Pescara, 66100 Chieti, Italy; 3 Department of Medicine and Aging Science, University “G. d’Annunzio” of Chieti-Pescara, 66100 Chieti, Italy; 4 Department of Pediatrics, “Spirito Santo” Hospital, 65100 Pescara, Italy; silvia.dimichele@ausl.pe.it; 5 Department of Maternal and Child Health, Neonatal Intensive Care Unit NICU, “Spirito Santo” Hospital, 65100 Pescara, Italy; paola.fusilli@ausl.pe.it; 6 Metabolic Diseases Unit, Bambino Gesù Children Hospital and Research Institute, 00165 Rome, Italy; giovanna.cotugno@opbg.net (G.C.); carlo.dionisivici@opbg.net (C.D.-V.); 7 Department of Psychological, Health and Territory Sciences, School of Medicine and Health Sciences, “G. d’Annunzio” University, 66100 Chieti, Italy

**Keywords:** phenylketonuria, hyperphenylalaninemia, inborn error of metabolism, mass spectrometry, metabolomics, LC–MS/MS

## Abstract

Phenylketonuria (PKU) is a rare autosomal recessive condition affecting about 1 in 10,000 people in the Europe, with a higher rate in some countries, like Ireland and Italy. In Italy, newborn screening (NBS) by MS/MS allows the diagnostic suspicion of PKU and its variants (Hyperphenylalaninemia (HPA), Tetrahydrobiopterin (BH4) synthesis deficiency, and Tetrahydrobiopterin (BH4) recycling deficiency) through the quantification of Phenylalanine (Phe) and the Phenylalanine/Tyrosine (Phe/Tyr) ratio in dried blood Spot (DBS) samples. Here, we report a case of an HPA whose suspicion was possible with expanded NBS, even if the normal-weight newborn was in total parenteral nutrition (TPN). It is known that TPN may present metabolic alterations, mainly for amino acids at NBS in MS/MS, frequently causing false positives. Actually, TPN is considered a special protocol in NBS, requiring several sample collections. In particular, a DBS sample is required before TPN, at basal time point (48 h after birth) and 72 h after the end of the procedure. In the case we report, even if the first DBS sample (before TPN) resulted negative, the repeated NBS tests revealed increased levels of Phe and dramatically high Phe/Tyr ratio. Thus, the newborn was recalled, and the NBS test was repeated several times before that HPA suspicion was confirmed by other specific biochemical tests. This case highlights the importance of Phe/Tyr ratio, only detectable by MS/MS analysis, in supporting the diagnostic suspicion during amino acids administration in the neonatal period.

## 1. Introduction

Hyperphenylalaninemia (HPA) are a group of inborn errors of metabolism characterized by accumulation of Phenylalanine (Phe) in blood and in other tissues, as a consequence of a defective phenylalanine hydroxylase (PAH) activity. Phe is converted in Tyrosine (Tyr) by PAH which requires the cofactor tetrahydrobiopterin (BH4), molecular oxygen, and iron for its activity. In most cases (about 98%), HPA results from mutations in the PAH gene [1]. The associated phenotypes range from classic phenylketonuria (PKU), the most severe form of the disease, to mild HPA forms. The remaining variants are caused by a block in the metabolism of the cofactor BH4, and in particular by BH4 synthesis deficiency and BH4 recycling deficiency [1,2]. HPA is the most frequent inherited disorder of amino acid metabolism, with a prevalence of 1:10,000 live-births in Europe, with a higher rate in some countries, such as Ireland and Italy. Nowadays, neonates affected by PKU or other variants of HPA are mostly identified in the neonatal period through newborn screening (NBS) programs. In particular, expanded NBS based on tandem mass spectrometry (MS/MS) allows the diagnostic suspicion through the quantification of Phe and the Phe/Tyr ratio in dried blood spot (DBS) samples. If untreated, alterations of Phe lead to progressive and irreversible mental disability, also accompanied by other symptoms, including seizures, eczematous rash, and motor deficit. Thus, the early recognition of the disorder through NBS and a prompt intervention followed by an immediate treatment, mainly through dietary restriction of Phe, allows us to avoid the neurological problems and the intellectual disability. In a single test, expanded NBS based on MS/MS allows a rapid and simultaneous quantification of several amino acids (AAs) and acylcarnitines (ACs) for the detection of more than 40 different inborn errors of metabolism (IEM), possibly when asymptomatic [3]. In quantifying metabolites by MS/MS analysis, many factors may influence levels of AAs and ACs, in NBS routine practice. In particular, levels of metabolites may vary, together with specific biological metabolic processes influenced by medical treatment or by external factors [4].

### Newborn Screening: Management Procedures of Newborns and Infants in Particular Conditions

Premature babies, as well as small-for-gestation-age babies, may present transient HPA, especially when feeding a parenteral nutrition with AAs during their hospitalization in neonatal intensive care unit. For these newborns, various medical therapies and the liver metabolic immaturity contribute to generate high rates of presumptive positive at expanded NBS. Total parenteral nutrition (TPN) is one of the fundamental medical therapies in the management of the preterm infants, to prevent nutritional deficits, since it provide nutrients, minerals, and vitamins for growth and development [5]. Therefore, prematurity, birth weight, transfusion, and parental nutrition could all potentially influence NBS results [3]. Thus, this population requires special protocols for collecting the NBS specimen in order to reduce the potential of false-positive results and additional confirmation tests when no real disorder exists. These special protocols provide repeated tests during the postnatal period for premature babies, for low-birth-weight babies, for term/preterm babies on TPN, and for infants after transfusion [3]. Considering the high risk of false-positive results, many NBS laboratory evaluate not only AAs concentrations as primary biomarkers, but also their ratios: leucine/alanine ratio for maple syrup urine disease, methionine/phenylalanine ratio for hypermethioninemia, and Phe/Tyr ratio for HPA [5]. In case of AAs and ratio abnormalities in newborns on TPN, follow-up is strongly recommended, until the end of the TPN procedure, and even after its term. Another complicating factor in expanded NBS based on MS/MS may be the detection of maternal deficiency. In this case, false positives at NBS may be due to maternal defects, resulting in transplacental passage of metabolites [3,6].

Here, we report a case of an HPA suspicion in a normal-weight newborn on TPN. Even if the first DBS sample (before TPN) resulted negative, the following NBS tests revealed an increased and abnormal value of Phe and dramatically high Phe/Tyr ratio. After repeated NBS test, the HPA suspicion was confirmed by further specific biochemical tests, until molecular testing. 

## 2. Case Report

### 2.1. Clinical Presentation

The male baby had been delivered by cesarean section at 41 weeks of gestation, with meconium-stained amniotic fluid. The parents were unrelated, and the healthy mother had never been exposed to any drugs during the uneventful pregnancy. The auxological parameters were appropriate to gestational age. Apgar score were 3, 6, and 8 at 1, 5, and 10 min, respectively. Due to meconium aspiration syndrome, the infant required resuscitation with intubation in the surgery room. The passive cooling for suspected perinatal asphyxia was also started. The cord arterial blood gas analysis revealed severe metabolic acidosis (pH of 6.85, base deficit of −23.9 mmol/L, bicarbonate of 11.6 mmol/L, and lactate 12 mmol/L) confirmed at the baby’s 30 min arterial blood gas analysis (pH of 7.14, base deficit of −16.1 mmol/L, bicarbonate of 13.1 mmol/L, and lactate 10.8 mmol/L). 

At the admission at the Neonatal Intensive Care Unit (NICU), the baby was treated with surfactant replacement and was placed on conventional mechanical ventilation and cardiovascular support (i.e., dopamine and dobutamine); the oxygenation index was 1.2, and there was no evidence of pulmonary hypertension. The passive cooling was stopped at first hour of life due to normal neurological examination and continuous normal voltage on amplitude-integrated electroencephalogram (aEEG) monitoring. 

Despite high level of C-reactive protein (195 mg/L), blood, bronchoalveolar lavage, and pharyngeal swab cultures were sterile, and the empirical antibiotic therapy (ampicillin-sulbactam and gentamicin) was discontinued on day six of life. 

Since improvement was observed on the respiratory support, the newborn was successfully extubated at day five and was supported on non-invasive ventilation for three more days.

The baby was fed intravenously with TPN in the first four days. Details of TPN composition (Tph, 6% solution for infusion, Baxter S.p.A.) are well reported in Supplementary Materials (Table S1). By the fifth day, he continued to be fed with mixed parenteral nutrition, starting to tolerate full breastmilk feed. 

Blood samples for NBS were obtained by heel stick, spotted on filter paper, dried at room temperature, and sent to the newborn screening laboratory. DBS sampling were performed on day 1, 2, 9, and 14 and revealed an elevated Phe level. Parenteral nutrition was stopped in the 14th day of life, and he was discharged in the 16th day with normal clinical and neurological findings and exclusively breastfed. His developmental monitoring and support were ongoing; in the follow-up visits, the patient presented in good health.

### 2.2. Newborn Screening Test

According to the laboratory collection protocol, samples for NBS were obtained before starting TPN (first hour of life), at 48 and 72 h after TPN discontinuation. Heel-prick blood specimen were spotted onto PerkinElmer collection device (Ahlstrom 226 filter paper). The DBS sample from the newborn was punched out into a final diameter disk of approximately 3.2 mm, using an automatic puncher, into five polypropylene microtiter plates for newborn screening testing: four immunofluorometric assays for congenital hypothyroidism, cystic fibrosis, galactosemia, and biotinidase deficiency, and a flow injection analysis–tandem mass spectrometry (FIA–MS/MS) for 36 further IEM among amino acid disorders, urea cycle disorders, organic acid disorders, and fatty acid oxidation disorders, according to the Italian practices of NBS. For expanded NBS test by FIA–MS/MS, a 3.2 mm DBS disk (equivalent to approximately 3–3.2 μL whole blood) was extracted for the determination of 14 AAs, 35 ACCs, C0, and succinylacetone. The internal standards, as well as the extraction solution, were obtained from the NeoBase 2Non-Derivatized MSMS Kit (Perkin Elmer Life and Analytical Sciences, Turku, Finland). Mass spectra were acquired in positive electrospray ionization, using multiple reaction monitoring (MRM) as acquisition mode, using an Ultra-Performance Liquid Chromatography/Tandem Quadrupole Mass Spectrometry (UPLC/MS/MS) system (Acquity UPLC I-Class coupled to a Xevo TQD, Waters Corp., Manchester, UK) [7,8,9]. Data were processed by MassLynx V4.2 and NeoLynx Software (Water Corp.). Then, 10 µL was injected into the ion source, and the run time was 1.1 min, injection-to-injection.

Since the newborn presented high levels of immunoreactive trypsinogen (IRT) at the first screening test, a genetic test was performed for CFTR gene, as required by our protocol. Genomic DNA was extracted from DBS by using MagPurix instrument and Forensic DNA extraction Kit (12sZinexts Life Science Corp.—CodZP01001) according to the manufacturer’s protocol. A multiplex PCR was carried out by using “Devyser CFTR Core”, which detects the most common mutations found in population of European origin, and “Devyser CFTR Italia”, which includes a panel supporting the detection of mutations specifically found in the Italian population (Devyser AB, Instrumentvägen 19. SE-12653 Hägersten, Sweden). Both genetic tests resulted in negative for the mutations analyzed.

**Figure 1:**
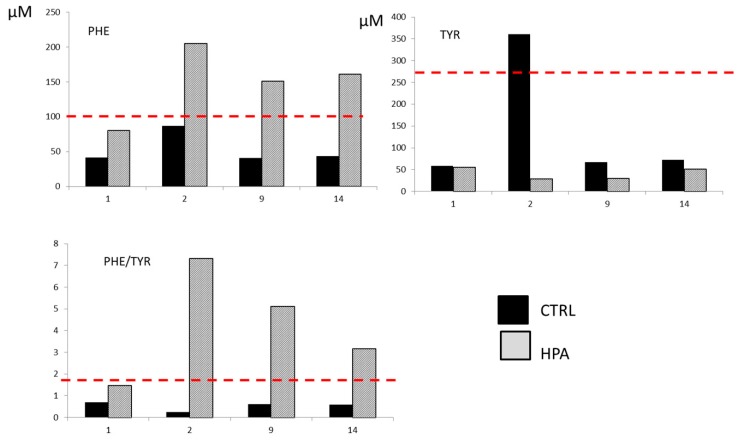
Alterations at expanded newborn screening (NBS). Histograms represent the levels of Phe, Tyr, and Phe/Tyr ratio at 1, 2, 9, and 14 days of life for HPA case (light-gray bar) and for a healthy control newborn (black bar). Red dashed line indicates cut-off limits for each analyte and for the ratio.

Metabolic profile of the newborn by FIA–MS/MS at the first test (before TPN, < 48 h) revealed Phe levels and Phe/Tyr ratio nearly cut-off: Phe = 80.4 µmol/L (normal value (n.v.) < 100 µmol/L) and Phe/Tyr= 1.46 (n.v. < 1.8). The following analysis during TPN at 2, 5, 9, and 14 days of life revealed an increase of Phe concentrations and of Phe/Tyr ratio, in particular, at the second day of life Phe = 205 µmol/L and Phe/Tyr = 7.32; at the 5th day of life Phe = 304 µmol/L and Phe/Tyr = 6.60; at the 9th day of life Phe = 151 µmol/L and Phe/Tyr = 5.10; and at the 14th day of life Phe = 161 µmol/L and Phe/Tyr= 3.15. Histograms in Figure 1 show the levels of Phe, Tyr, and Phe/Tyr ratio for the HPA case (light gray) and a ctrl subject (black bar) before the parental nutrition (first hour of life) and during the time points of the procedure. As shown, the HPA case is just below the cut-off for all analytes before TPN, while the HPA diagnostic markers are above the cut-off during the procedure. In the following tests, at the end of the TPN procedure and during the follow-up (monthly checked), the Phe levels and Phe/Tyr ratio were constantly altered, 143 µmol/L and 2.95, respectively. In order to evaluate the metabolic alterations, further analyses for diagnostic confirmation were performed.

### 2.3. Hyperphenylalaninemia Diagnostic Confirmations 

The infant continued to be followed for HPA suspicion. The first evaluation of plasma AAs also revealed an increase in the levels of Phe (Phe = 327 µmol/L, n.v.: 30–80 µmol/L). Moreover, the diagnostic confirmation continued with the determination of urinary pterines showing normal profile at neopterin/biopterin urinary test. Echo brain scan was normal. The infant continued free diet for age with breastfeeding and formula, showing a good increase in weight and length until two months. At the age of two months, the infant began to present gastroesophageal reflux, so he started an antacid therapy and a hydrolyzed milk (Pregominsp). At the age of six months, the neurological examination and the evaluation of development (Bayley scale) were normal for age. The HPA was finally confirmed as compound heterozygous by molecular analysis. The mutations found were p.Phe39del and p.Ala403Val corresponding to the following genotypes: c.[115_117delTTC] and [1208C > T]. The segregation was also confirmed in the parents. In particular, the molecular analysis of his father and his mother has documented the following mutations in heterozygosity: p.Phe39del (c.115_117delTTC) andp.Ala403Val (c.1208C > T), respectively. 

## 3. Discussion

Expanded NBS through targeted metabolomic approaches has led significant advantages in terms of diagnosis and treatment of IEM, even if it also brought out a number of “side effects”. Considering that metabolomic findings are influenced not only by genome, but also by diet, drugs, maternal defects, and external factors, there are many unintended consequences of expanded NBS, such as overdiagnosis, unnecessary treatment, and parental anxiety [10]. Thus, metabolic alterations at NBS may be related to an IEM, to partial enzyme deficiencies, to maternal defects, and to other factors such as prematurity, parental nutrition, therapies, nutritional deficiency, and a sick infant. In Italy, as in other countries, blood collection for NBS is recommended between 48 and 72 h of life [3]. In most cases, treatments such as TPN start within the 24 h of life, leading to metabolic profiling alterations at NBS. Parental nutrition is a solution rich in AAs, thus newborns on TPN may show multiple minor AAs abnormalities at NBS. Higher Phe levels are reported in very premature infants on TPN [11]. In our experience, we observed alterations in AAs levels, such as Arginine, Methionine, branched chain AAs, Phe, and Tyr. The simultaneous increase of such AAs often generates a normal value of AAs ratio. In particular, in case of HPA or PKU suspicion, both the alterations of Phe and Phe/Tyr ratio should be revealed, as already discussed [12,13,14]. Actually, the evaluation of Phe/Tyr ratio has already been described as able to detect affected newborns without increasing the rate of false positives [12], to avoid false negatives [14], and to act as a powerful diagnostic tool in differential diagnosis of HPA variants [15]. Moreover, the importance of the Phe/Tyr ratio has been highlighted in its ability to detect the disease, even with the specimen collected at 4 h after birth [14]. Even if Phe/Tyr ratio is not considered a primary marker for PKU and HPA, this ratio became really significant and informative in some special condition for example babies on TPN. Furthermore, we would like to emphasize the importance of the Phe/Tyr ratio not only for TPN protocols during the neonatal period, but also for the long-term follow-up of HPA patients. Increased level of Phe/Tyr ratio is recently recognized as a sensitive indicator of the metabolic state in HPA patients, being strongly associated with cognitive deficits, often more than the increase of Phe level alone [16]. In addition, it is crucial the evaluation of the Phe/Tyr ratio when the follow-up is performed with a different method, such as an AA analyzer. Due to the considerable method variability, a single method is suggested for the long-term follow-up of patients. For this reason, it is strongly recommended to inform clinicians about the method used in the monitoring of HPA patients [16].

On the presented case, Phe/Tyr alteration during TPN was exponentially high if compared with the increase of AAs, thus suggesting an enzyme defect suspicion more than the primary marker. Figure 1 shows a healthy control newborn (not HPA) before and during TPN, which never revels alteration of Phe/Tyr ratio, while an increase in the levels of Tyr and Phe (nearly cut-off value) were reported when starting TPN (day 2). Moreover, at the first NBS, the patient case showed nearly cut-off Phe levels, which is unusual for full-term unfed newborns. Although very few cases of newborns in TPN affected with IEM are reported, early suspicion of PKU/HPA in such newborns is noteworthy, since the AA intake can lead to a higher and faster increase of Phe compared to breastfeeding [17,18]. Reduced Phe TPN solutions should be used as soon as the suspicion is confirmed.

These data deeply confirm the importance of repeated NBS tests in the case of particular conditions. In fact, many are the factors influencing metabolic profile, as well as the special condition (prematurity and low birth weight) may be the effect of an IEM. Interestingly, an investigation by Mandouret. et al. already showed the longitudinal changes in AA and acylcarnitine profiles of preterm neonates over the first two weeks of life, indicating an age-related distribution of their concentrations, and pointing out the importance of using appropriate reference values when working with a prematurely born population [19].Thus, special protocols, which provide repeated tests for premature babies, for small-for-gestation-age babies, for term/preterm babies on TPN, and for infants after transfusion in the postnatal period, are required in order to reduce false-positive results and the additional confirmatory testing when not really necessary, but also to avoid false-negative results. As the best strategy to monitor the metabolic status of term/preterm babies on TPN, it is recommended to collect the first sample before TPN procedure, a second sample between 48 and 72 h of life, and the last sample at 72 h after the ending of parental nutrition [3].

Actually, with the present clinical case, we would like to emphasize the importance of marker ratios, as Phe/Tyr ratio, by MS/MS in supporting the HPA diagnostic suspicion during the administration of parental nutrition rich in AAs in the neonatal period. This case shows as many biomarkers may better describe a metabolic disease than a single marker can do, perfectly fitting with the “omics” and the “personalized medicine” era. This era presents a real perspective to further expand NBS to many other metabolites and disorders, improving the metabolic fingerprint description in newborns. Taking into consideration all these perspectives, the use of post-analytical tools in expanded NBS should be strongly suggested since the simultaneous evaluation of many metabolites may result in being difficult. These post-analytical tools, such as Region 4 Stork (R4S) and the more recent Collaborative Laboratory Integrated Reports (CLIR, https://clir.mayo.edu), are meant to improve the interpretation of the complex metabolite profile and to aid in the differential diagnosis of abnormal results, also reducing the possibility of false-positive and/or false-negative results [20,21]. In fact, a critical point of expanded NBS is to limit or better avoid the occurrence of such errors, sometimes arisen from a rigid application of cut-off values, mainly when further informative markers and analyte ratios are not evaluated [20].

## Supplementary Materials

The following are available online at https://www.mdpi.com/2218-1989/10/2/44/s1. Table S1: The active ingredients of TPN solution.
