# Supplementary material for: A Case of Suspected Hyperphenylalaninemia at Newborn Screening by Tandem Mass Spectrometry during Total Parenteral Nutrition
Source: Metabolites. 2020 Jan 24;10(2):44. doi: 10.3390/metabo10020044 (PMC7074497; doi:10.3390/metabo10020044)
Supplement: Supplementary file 1 [file metabolites-10-00044-s001.pdf]

metabolites

Table S1. The active ingredients of TPN solution.

Essential Amino Acids (g)		Non-essential Amino Acids (g)	
**L-Phenylalanine**	**2.9**	L-Alanine	3.2
L-Isoleucine	4.9	L-Arginine	7.3
L-Leucine	8.4	L-Proline	4.1
L-Lysine (*)	4.9	L-Serine	2.3
L-Methionine	2.0	Glycine	2.2
L-Threonine	2.5	L-aspartic acid	1.9
L-Tryptophan	1.2	Glutamic acid	3.0
L-Valine	4.7		
L-histidin	2.9		
L-Cysteine.HCI.H2O	0.2		
Taurine	0.15		
L-Tyrosine (**)	1.4		

Table lists the ingredients contained per 1000 ml of solution Thp, 6% solution for infusion, Baxter S.p.A. (*) Added as lysine acetate 6.9 g/ l. (**) As L-Tyrosine 0.4 g and N-acetyl-Tyrosine 1.2g.
